# Depersonalization-Derealization Disorder: Etiological Mechanism, Diagnosis and Management

**DOI:** 10.15190/d.2024.09

**Published:** 2024-06-30

**Authors:** Harsahaj Singh Wilkhoo, Afra Wasama Islam, Felcia Reji, Labdhi Sanghvi, Rhea Potdar, Siddhant Solanki

**Affiliations:** ^1^Faculty of Medicine, Tbilisi State Medical University, Georgia; ^2^Mahatma Gandhi Institute of Medical Sciences, India; ^3^Tbilisi State Medical University, Georgia

**Keywords:** Derealization-depersonalization disorder, Neurology, Psychiatry, Dissociative disorder.

## Abstract

This comprehensive review delves into the complexities surrounding Depersonalization-Derealization disorder (DPDR), a dissociative disorder characterized by enduring feelings of detachment from one's self and surroundings. Tracing its historical roots back to 19th-century descriptions and its current classification as a singular disorder, the review meticulously explores the clinical presentation, epidemiology, etiology, diagnosis, and management of DPDR.  Despite many trials and studies conducted the exact cause of this condition is still unknown. The best way to understand its etiology is by taking into account its clinical presentations and linking it to different structural and functional alterations of the brain. Alteration in cortical activity and structure associated with white matter, gray matter, caudate nucleus, amygdala, and other areas like Broadman’s areas of cortex are analyzed to be potential mechanisms for etiology. With a concerning rise in its prevalence globally and notable impact on adolescents and young adults, DPDR manifests through a spectrum of symptoms including depersonalization, and derealization, and often accompanies comorbidities such as anxiety and depression. While the precise cause remains elusive, factors such as traumatic experiences, stress, and genetic predispositions have been implicated, with modern neuroimaging studies offering insight into potential structural and functional brain alterations. Managing DPDR necessitates a multifaceted approach integrating psychotherapy, pharmacotherapy, and lifestyle interventions, with cognitive-behavioral therapy (CBT) and pharmacological agents like SSRIs and SNRIs emerging as primary interventions. The importance of early detection and intervention is crucial for improving its prognosis. Unfortunately, DPDR is highly understudied to date. Due to a scarcity of scientific literature about DPDR in recent years, it has become very challenging to get a proper in-depth understanding of this. Therefore, this review serves as an all-in-one source to get information about DPDR ranging from etiology to its management strategies.

## SUMMARY

1. Introduction

2. Definition

3. Clinical presentation

4. Epidemiology

- 4.1 Prevalence of DPDR

- 4.2 Association of comorbid condition

- 4.3 Self-assessment by patient and misdiagnosis

5. Etiology

- 5.1 What causes DPDR

- 5.2 Response to trauma

- 5.3 Co-existing or associated disorder

- 5.4 Neuroanatomical and neurobiological perspective

6. Diagnosis

- 6.1 Diagnosis methods and approaches

- 6.2 Differential diagnosis

7. Management

- 7.1 Therapeutic approaches

- 7.2 Pharmacologic intervention

- 7.3 Lifestyle Modification and Support

- 7.4 Long-term prognosis and personalized management approaches

8. Conclusion 

## 1. Introduction

Derealization (DR) and depersonalization (DP) are symptoms categorized as feelings of unreality and detachment from own self and surroundings. Deperzonalization-derealization disorder (DPDR), formerly classified as a separate phenomenon, now is a single dissociative disorder characterized by persistent and distressing episodes with functional impairment, depicting it as a clinical diagnosis that can occur alone or in conjunction with other mental disorders^^1^^. It’s a very under-researched topic and was first described in the 19th century^^2,3^^. Ever since there have been many descriptions of this condition^^4^^. The prevalence rate of DPDR ranges from 1 to 2%, being higher in adolescents and young adults^^5^^. However, certain studies have shown a prevalence of potential symptoms of about 20%^^6,7^^. The frequent feelings of disembodiment and emotional numbness that may extend beyond the present moment to include memories and imagination or a weakened ability to respond to emotional circumstances, although the capacity for emotional expression and reality testing remains intact^^8^^. It can also be accompanied by other mental disorders such as anxiety, and schizophrenia, as well as difficulties in concentration and memory retrieval, which can profoundly affect the quality of life of patients and interfere with their daily activities and social relationships^^9,10^^.

Although the exact cause of DPDR is yet to be known, traumatic experiences and childhood anxiety are understood to be common triggers. Other triggers include intense stress, depression, panic attacks, and ingestion of psychoactive substances^^10^^. Several neurotransmitter systems have been implicated; including classes of chemicals such as glutamate NMDA receptor antagonists, cannabinoids, hallucinogens, and opioid receptor agonists; although evidence for each is scant and partly indirect. Research on the diagnosis, cause, and treatment of DPDR is an urgent need due to the rise in mental disorders along with urbanization. Prompt diagnosis and early treatment can save a patient from a life-long debilitating disease that places them as a burden on themselves and their family. This review article aims to provide a detailed and updated perspective of Depersonalization-Derealization disorder (DPDR) highlighting its etiology, epidemiology, key diagnostic findings as well as correlation with other disorders^^11^^. This review also gives information on management techniques and advances in management as well as prognosis.

## 2. Definition

Depersonalization-derealization disorder, as listed in ICD 11 under the code 6B66 and code F48.1 in ICD 10 respectively is a rare condition characterized by persistent or recurrent experience of depersonalization or derealization or both^^12^^. Depersonalization is defined as an alteration of perception and a feeling of the self as strange or unreal, or by feeling detached from, or as an outside observer of, one's thoughts, feelings, sensations, body, or actions. Derealization is defined as the perception of other people, objects, or the world as strange or unreal. Patients frequently express feelings of estrangement or detachment from their thoughts, bodies, or the real world^^13^^. It’s the alteration in the perception of the world so that it seems unreal as defined by the American Psychiatric Association^^14^^. Refer to Table 1 for a comparison.

**Table 1:** Table 1. Comparison of concepts^12-14^

A	B	C
Derealization	Depersonalization	DPDR
Perception of other people, objects, or the world as strange or unreal. Feelings of estrangement or detachment from their thoughts, bodies, or the real world	Alteration of perception and a feeling of the self as strange or unreal, or by feeling detached from, or as an outside observer of, one's thoughts, feelings, sensations, body, or actions.	Persistent experience of depersonalization or derealization or both Often presented as a co-existing condition for a longer period of time.

## 3. Clinical Presentation

**Figure 1:**
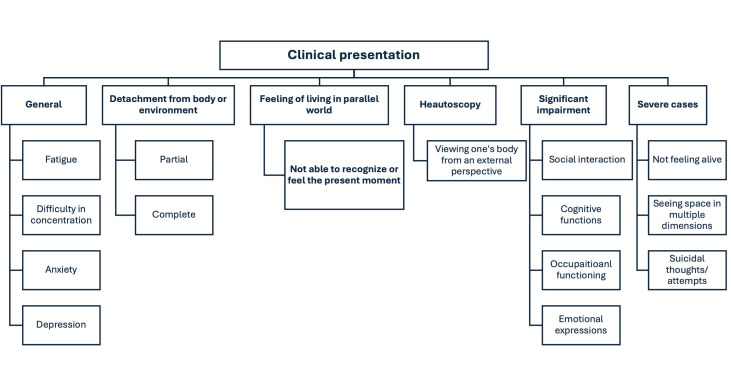
FIgure 1. Main clinical presentation of a patient with derealization depersonalization disorder

Although there is no proper description of what a person feels in this condition, people experiencing derealization feel that their perception of the situation or the current state is unreal^^15^^. Patients with DPDR may present with one or two or a combination of symptoms of derealization or depersonalization along with some other general symptoms as other psychiatric disorders like depression, and anxiety disorders as illustrated in Figure 1. Due to these reasons, proper differential diagnosis is crucial. It is often seen as a combination of symptoms. They describe not being themselves and experiencing a sense of detachment from their body or the environment partially or completely. Some patients have also reported symptoms like heautoscopy (autoscopy) which means seeing one’s body from an external perspective^^8,15^^. While other patients describe their experience as living in a parallel world without being the actor in their life. It can lead to a weakened ability to respond to emotional circumstances, however, the capacity for emotional expression and reality testing remains intact^^13^^. It could be as severe as feeling not being alive or seeing space in multiple dimensions. This sense of unreality about the outside world is believed to be a defense mechanism of the person’s brain to protect them from any traumatic situation or any kind of anxiety that can arise^^16^^. Acute cases of derealization depersonalization disorder can arise due to some conditions such as fatigue, sleep deprivation, or even traveling to an unfamiliar place^^10,17^^. The symptoms result in significant distress or impairment in personal, family, social, educational, occupational, or other important areas of functioning. Generally, patients with Depersonalization-Derealization disorder may also present with anxiety, fatigue, insomnia, and depression-like symptoms^^10,18^^. But when derealization depersonalization is chronic, it is considered a dissociative condition, depersonalization derealization disorder (DPDR).A person with this condition may experience an inability or difficulty in recognition of their reflection, diminished or complete loss of sensation

in parts of the body, feeling that the general life events are unreal or are happening in their dreams, or at times it can also affect memory resulting in a person feeling that their known ones are strangers and the places that they are very familiar to feel strange and unknown.

## 4. Epidemiology

### 4.1 Prevalence of DPDR

Depersonalization-derealization disorder is diagnosed in about 2% of the population and there is no gender prevalence as it occurs in both males and females equally. The symptoms of depersonalization-derealization disorder vary from person to person and can sometimes be asymptomatic as well^^1^^. The lifetime prevalence of the transient symptoms in the general population is studied to be about 70% and around 66% during a traumatic event^^5,19^^. Higher prevalence rates are seen in patients with specific psychiatric disorders like panic disorders, PTSD, and depression, with the rates going as high as 80-85^^19^^. The earliest age of onset of DPDR has been reported to be 16 years old usually before the age of 25 but cases have also been reported in the early and late 20s, 30s, and 40s^^18,20^^.

**Table 2:** Table 2. Epidemiology of DPDR Note: Table derived by information obtained from “Michal M, Adler J, Wiltink J, Reiner I, Tschan R, Wölfling K, et al. A case series of 223 patients with depersonalization-derealization disorder. BMC Psychiatry. 2016”

Statistic	Value
Prevalence in the general population	2%
Gender prevalence	Equal in males and females
Lifetime prevalence of transient symptoms	70%
Prevalence during a traumatic incident	66%
Prevalence in specific psychiatric disorders	80-85%
Earliest age of onset detected	16 years
The common age of onset	Before 25 but can be reported in 30s, 40s and 50s.
Regional higher prevalence	United Kingdom, Spain, Colombia, Mexico, Switzerland
Contributing factor	Higher anxiety levels in adolescents
Genetic influence	48%
Environmental risk, the parent with anxiety disorders	95%
Differential diagnosis challenges	Often mistaken for anxiety, depression, or personality disorder
Common misassumptions by patients	Tumor in brain, eye disease, drug-induced brain damage
Common co-morbid conditions	Substance abuse disorders, dissociative spectrum disorders, anxiety disorders, borderline personality disorders, schizophrenia, depression

Although males are majorly dominant where most of the cases have been reported, there also have been reports of the sex ratio as 1:1 which may last for several months, years, or a day or two^^18^^. Sadly, our research studies have found no prevalent ethnicity, but rather that this affects 2% of people all over the world. However, there are certain regions in the world where cases are more prevalent rather than others, these regions are the United Kingdom, Spain, Colombia, Mexico, and Switzerland. Our analysis also revealed that higher levels of anxiety in adolescents could be a contributing factor to explaining the higher number of DPDR in the younger population^^5^^.

### 4.2 Association with co-morbid conditions

Moreover, studies into the biography of patients diagnosed with this disorder have also revealed an ancestry of various forms of anxiety disorders, psychiatric and dissociative disorders which may also lead to an increased genetic susceptibility on one side and the other side to a higher environmental risk of being exposed to parents with anxiety disorders in 95% of the cases^^18^^. Studies also reported a genetic influence of around 48%^^20^^.

### 4.3 Self-assessment by patient and misdiagnosis

After the initial discovery of symptoms, patients would often learn about their diagnosis through the web or social networks and social networks; however, this would be neglected in the beginning due to the lack of knowledge of different physicians and would often be diagnosed as some form of anxiety, depressive or personality disorder^^18^^. Rarely, patients may assume this as a form of tumor in the brain, eye disease, or drug-induced brain damage as the cause of symptoms and would visit a neurologist, ophthalmologist, or other specialties before visiting a psychiatrist or psychologist. All the above-mentioned epidemiologic factors and their distribution are summarized in Table 2.

## 5. Etiology

### 5.1 What causes DPDR?

So far there is no proper etiology that has been stated for DPDR. However, with the help of clinical associations of the symptoms and other associated conditions, potential etiology can be understood^^3,21^^. There is no specific age of onset or the pace at which this condition progresses. However, studies have shown the most common age to be late 20s^^3^^. Among the most widespread causes of patients with DPDR some of the frequent ones are a family history of anxiety disorders, daily life stress events, COVID-19, parental divorce, personal relationship issues, interpersonal conflicts, and less common episodes associated with acute onset including a drug history involving cocaine, amphetamines, ecstasy, cannabis, and Ritalin among many others^^22,23^^. Some of the patients with “Long COVID” also have reported conditions like brain fog and depersonalization derealization. The exact etiology is unknown. However, certain studies suggest that the reduction of serotonin levels due to viral persistence may be a reason for these symptoms in long COVID^^24^^. There have also been many reports that childhood trauma treatment such as physical, mental, and emotional abuse can be associated with severe types of PTSD and stress disorders^^18^^. Significantly, our research also found that higher levels of emotional abuse and lower levels of physical abuse were associated with increased depersonalization and derealization.

### 5.2 Response to trauma

In most cases, depersonalization and derealization are seen as a response to a traumatic incident and occur simultaneously in someone's life, and the terms individually such as depersonalization refer to

**Figure 2:**
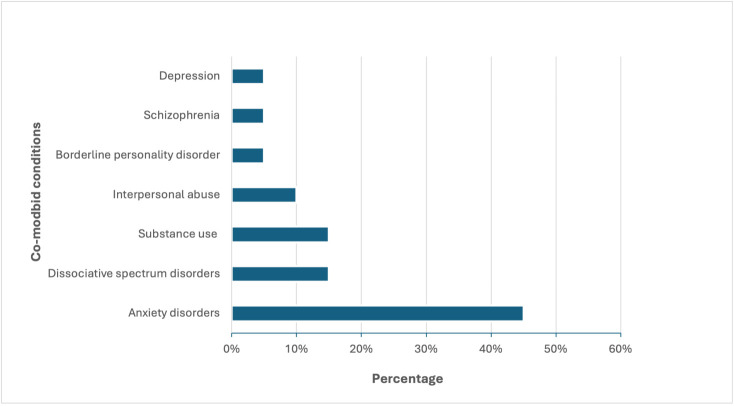
Figure 2. Chart illustrating commonly reported comorbid conditions with DPDR. Derived from reproducing information from references^16-18^

detachment from your identity and viewing your behavior, emotions, and bodily sensations as someone from a distance removed and, on the other hand, derealization refers to the feeling that the world around you is dead, deserted, dreamy, or distorted and disturbed^^16,17^^.

### 5.3 Co-existing or associated disorders

Some of the most reported comorbidities are anxiety disorders (45%), dissociative spectrum disorders (15%), substance use disorders (15%), interpersonal abuse (10%), borderline personality disorder (5%), schizophrenia or schizophrenia spectrum disorders (5%) and depression (5%) as illustrated in Figure 2. Furthermore, DPDR has also reported a strong association with Axis I disorders along with Axis II disorders (60%); these include not only personality disorders but also various obsessive-compulsive disorders along with borderline and avoidant disorders^^18^^.

### 5.4 Neuroanatomical and neurobiological perspective

With the advancements in neuroimaging technologies, studies have revealed a potential association of functional and structural alteration with DPDR^^8^^. White matter is one important structure that is studied for the same. In patients with DPDR, these connections between the brain regions are altered^^25^^. These alterations can be primarily as a pathophysiology or can be secondarily linked to local gray matter loss. Both of these can give rise to such symptoms^^8^^. Among many types of magnetic resonance imaging (MRI) techniques, functional and structural MRI have shown us these alterations in various brain regions^^26-28^^.

A study done on drug naïve patients showed a correlation between fractional anisotropy (FA) of the abnormal white matter fiber with cognitive impairments having DPDR symptoms. A higher FA in the right corpus callosum and posterior corona radiata was observed ass compared to the otherwise healthy individuals. These showed a positive correlation with the CDS-30 scores giving rise to conditions like unreality, numbing, alterations in perception, and other symptoms linked to DPDR^^29^^. Other studies have also found a lower FA in left temporal and right temporoparietal regions in individuals with DPDR as compared to healthy individuals. These links were also associated with similar symptoms^^8^^. According to older understanding, DPDR was seen as a brain response against certain life-threatening conditions. Recent studies indicate a potential relation to temporal lobe dysfunction^^30^^. Certain studies have also indicated that left hemispheric frontotemporal activation is involved in the development of DPDR. According to the study, the depersonalization of epileptic origin has a strong association with frontal lobe epilepsy^^31^^. Interestingly, a PET scan-based study on depersonalization disorder revealed an association of Broadman’s areas of the cortex. As compared to the healthy individuals, there was a significantly lower activity in the right Broadman’s areas 22 and 21 of the superior and medial temporal gyri and a relatively higher metabolism in the Broadman’s area 7B and 39 of the parietal lobe as well as Broadman’s area 19 of the occipital lobe^^32^^.

Likewise, a study done on higher-risk patients found more prevalence of DPDR. Neuroimaging investigations have indicated a pathophysiological mechanism like a decrease in activity in the orbitofrontal cortex and an increase in activity of the caudate nucleus. These mechanisms result in a relatively common pathway leading to similar symptoms as DPDR thus acting as a potential mechanism for the progression and development of DPDR^^33^^.

The exact neurobiological mechanism remains unclear to date. However, the evidence from various studies as mentioned above indicates a significant association between brain regions involved in emotional perception and memory. Alterations in activities of brain areas like the amygdala, hippocampus, and middle/superior temporal gyrus along with white matter and other cortical regions are linked with DPDR^^34^^.

## 6. Diagnosis

### 6.1 Diagnostic methods and approaches

Diagnosis of DPDR is made based on a doctor’s evaluation of specific diagnostic criteria from the Diagnostic and Statistical Manual of Mental Disorders, Fifth Edition, Text Revision (DSM-5-TR) alongside certain tests to rule out other possible causes^^35^^. According to the Diagnostic and Statistical Manual of Mental Disorders (DSM-V), the diagnostic criteria for DPDR includes:

1. The existence of ongoing or recurring depersonalization, derealization, or both. Depersonalization: Perceptual distortions, a warped sense of time, an unreal or absent self, emotional and/or physical detachment, feelings of unreality, or being an outside observer with regard to one's thoughts, feelings, sensations, body, or actions or actual numbness). Derealization: Perceptions of unreality or disassociation from the environment (people or things seeming surreal, dreamlike, hazy, lifeless, or distorted in the eyes).

2. Reality testing is unaffected by the depersonalization or derealization experiences.

3. The symptoms impair social, occupational, or other critical domains of functioning or cause clinically significant distress.

4. The disruption cannot be attributed to a substance's physiological effects (such as drug misuse, prescription drugs), or other illness (e.g., seizures).

5. No other mental illness, such as schizophrenia, panic disorder, major depressive disorder, acute stress disorder, post-traumatic stress disorder, or other dissociative disorder, provides a better explanation for the disturbance.

Patients with depersonalization derealization disorder may find it difficult to articulate their symptoms and may believe that they are losing their minds. An additional prevalent feeling is the worry of permanent brain impairment. Subjectively altered sense of time (too fast or too slow) and subjective difficulty in vividly recalling memories and owning them as personal and emotional are commonly associated symptoms. They also experience vague somatic symptoms such as tingling, lightheadedness, or fullness in the head. Extreme rumination or obsessional preoccupation can occur in some people. Another common feature that is often present is varying degrees of anxiety and depression. Due to the fact that such comorbidities may exist, an accurate diagnosis is essential. It has been discovered that physiological hyperreactivity to emotional stimuli occurs in those who have the disorder. The inferior parietal lobule, the prefrontal cortical-limbic circuits, and the hypothalamic-pituitary-adrenocortical axis are among the neural substrates of interest^^1^^.

### 6.2 Differential diagnosis

To rule out other disorders, such as mental health disorders, seizure disorders, and substance use disorders, that could be the cause of symptoms, a physical examination and sometimes tests are performed. To detect the use of illegal drugs, tests may include blood and urine tests. Other common tests include magnetic resonance imaging (MRI), computed tomography (CT), and electroencephalography (EEG). Psychological tests and specially structured interviews and questionnaires can also help doctors with the diagnosis^^36^^. The primary motive of differential diagnosis is to rule out any psychiatric or neurological disorder which might also present with the same symptoms. Only after ruling out such factors, we can confirm DPDR. As mentioned above, there is no proper diagnostic method for diagnosing DPDR. With the help of etiological changes in neurobiology, we can develop a more accurate confirmatory diagnostic criteria for DPDR through neuroimaging. Future studies should focus on getting a deeper understanding of the same.

## 7. Management

### 7.1 Therapeutic approaches

Depersonalization and derealization disorder require a multifaceted approach encompassing therapeutic, pharmacological, and lifestyle interventions. Psychotherapy is the cornerstone of treatment in the absence of significant comorbid symptoms such as anxiety or depression. Cognitive-behavioral therapy (CBT) is the first-line treatment over other psychotherapies for most newly diagnosed patients, owing to its effectiveness in altering detrimental thought patterns and behaviors^^37^^. Dialectical behavior therapy (DBT) is also integral, particularly due to its role in emotion regulation and stress mitigation.

### 7.2 Pharmacologic intervention

Pharmacologically, selective serotonin reuptake inhibitors (SSRIs) and serotonin-norepinephrine reuptake inhibitors (SNRIs) are prevalently used, especially when DPDR is accompanied by anxiety or depressive disorders. In select cases, judicious use of low-dose antipsychotics or antianxiety medications may be warranted^^38^^.

### 7.3 Lifestyle modification and support

The management of DPDR extends beyond clinical interventions to include regular psychotherapeutic participation, stress reduction techniques, and active participation in support groups. Adopting a healthy lifestyle, characterized by regular physical activity, adequate sleep, and balanced nutrition, is essential for the holistic management of mental health^^39^^.

### 7.4 Long-term prognosis and personalized management approaches

Although a definitive cure for DPDR remains elusive, a substantial number of patients report considerable relief of symptoms through personalized customized treatment strategies. The emphasis is on managing symptoms effectively to enhance life quality. Preventive measures for DPDR are not explicitly defined due to its ambiguous causation. However, early intervention in stressful or traumatic scenarios, coupled with maintaining sound mental health and addressing symptoms promptly, is recommended for mitigation^^18^^. The prognosis for DPDR varies; episodic experiences in some contrast with more persistent symptomatology in others. The prognosis tends to be more optimistic with prompt and consistent treatment, particularly when concurrent mental health conditions are addressed effectively^^40^^. Addressing depersonalization and derealization disorder requires an integrative strategy, involving targeted psychotherapy, appropriate medication, and lifestyle modifications. Early detection and individualized treatment plans are pivotal in improving patient outcomes, underscoring the need for continued research and a deeper understanding of the disorder.

## 8. Conclusion

The understanding and management of Depersonalization-Derealization disorder (DPDR) present ongoing challenges in the field of healthcare. Through this comprehensive review, we have delved into the fine details of this disorder, from its clinical presentation to its epidemiology, etiology, diagnosis, and management. DPDR, once considered a disparate phenomenon, is now recognized as a singular dissociative disorder, characterized by persistent feelings of unreality and detachment from self and surroundings. Its prevalence, ranging from 1% to 2%, underscores the need for increased awareness and research on its causative factors and treatment modalities. The multifaceted nature of DPDR requires a holistic approach to treatment that incorporates psychotherapeutic, pharmacological, and lifestyle interventions. From cognitive-behavioral therapy to pharmacological agents like SSRIs and SNRIs, tailored treatment strategies aim to alleviate symptoms and improve the quality of life for affected individuals. Furthermore, recognition of comorbidities, such as anxiety and depression, underscores the importance of integrated care. As we navigate through the complexities of DPDR, early detection, and intervention emerge as crucial factors in shaping prognosis and enhancing patient outcomes. The major challenge faced during this study is the lack of scientific literature on this condition. DPDR, being an understudied condition, makes it difficult for researchers to get a proper understanding and identification of it. There is a lack of evidence for diagnosis, especially when it comes to radiographic imaging. We believe that future studies should have a detailed understanding of the characteristic radiologic diagnosis as well as the precise changes in the brain chemicals to have a proper diagnosis of DPDR.

## References

[1] American Psychiatric Association. Diagnostic and Statistical Manual of Mental Disorders: Fifth Edition Text Revision DSM-5-TRTM.

[2] Sierra Mauricio, Berrios German E. (2000). The Cambridge Depersonalisation Scale: a new instrument for the measurement of depersonalisation. Psychiatry Research.

[3] BAKER D., HUNTER E., LAWRENCE E., MEDFORD N., PATEL M., SENIOR C., SIERRA M., LAMBERT M. V., PHILLIPS M. L., DAVID A. S. (2003). Depersonalisation disorder: clinical features of 204 cases. The British Journal of Psychiatry.

[4] Shorvon H. J. (1946). The Depersonalization Syndrome. Proceedings of the Royal Society of Medicine.

[5] Yang Jinyan, Millman L. S. Merritt, David Anthony S., Hunter Elaine C.M. (2022). The Prevalence of Depersonalization-Derealization Disorder: A Systematic Review. Journal of Trauma &amp; Dissociation.

[6] Aderibigbe Y. A., Bloch R. M., Walker W. R. (2001). Prevalence of depersonalization and derealization experiences in a rural population. Social Psychiatry and Psychiatric Epidemiology.

[7] Kihlstrom John F., Glisky Martha L., Angiulo Michael J. (1994). Dissociative tendencies and dissociative disorders.. Journal of Abnormal Psychology.

[8] Sierk Anika, Daniels Judith K, Manthey Antje, Kok Jelmer G, Leemans Alexander, Gaebler Michael, Lamke Jan-Peter, Kruschwitz Johann, Walter Henrik (2018). White matter network alterations in patients with depersonalization/derealization disorder.. Journal of psychiatry & neuroscience : JPN.

[9] Lambert M V, Senior C, Fewtrell W D, Phillips M L, David A S (2001). Primary and secondary depersonalisation disorder: a psychometric study.. Journal of affective disorders.

[10] Salami Abbas, Andreu-Perez Javier, Gillmeister Helge (2020). Symptoms of depersonalisation/derealisation disorder as measured by brain electrical activity: A systematic review.. Neuroscience and biobehavioral reviews.

[11] Sierra Mauricio (2008). Depersonalization disorder: pharmacological approaches.. Expert review of neurotherapeutics.

[12] Reed Geoffrey M, First Michael B, Kogan Cary S, Hyman Steven E, Gureje Oye, Gaebel Wolfgang, Maj Mario, Stein Dan J, Maercker Andreas, Tyrer Peter, Claudino Angelica, Garralda Elena, Salvador-Carulla Luis, Ray Rajat, Saunders John B, Dua Tarun, Poznyak Vladimir, Medina-Mora María Elena, Pike Kathleen M, Ayuso-Mateos José L, Kanba Shigenobu, Keeley Jared W, Khoury Brigitte, Krasnov Valery N, Kulygina Maya, Lovell Anne M, de Jesus Mari Jair, Maruta Toshimasa, Matsumoto Chihiro, Rebello Tahilia J, Roberts Michael C, Robles Rebeca, Sharan Pratap, Zhao Min, Jablensky Assen, Udomratn Pichet, Rahimi-Movaghar Afarin, Rydelius Per-Anders, Bährer-Kohler Sabine, Watts Ann D, Saxena Shekhar (2019). Innovations and changes in the ICD-11 classification of mental, behavioural and neurodevelopmental disorders.. World psychiatry : official journal of the World Psychiatric Association (WPA).

[13] Sheaffer Heather Depersonalization and Derealization Disorder. Therapedia.

[14] (1994). Diagnostic A. Statistical Manual of mental disorders.

[15] Sar Vedat, Alioğlu Firdevs, Akyuz Gamze (2016). Depersonalization and derealization in self-report and clinical interview: The spectrum of borderline personality disorder, dissociative disorders, and healthy controls. Journal of Trauma &amp; Dissociation.

[16] Vannikov-Lugassi Miriam, Soffer-Dudek Nirit (2018). No Time Like the Present: Thinking About the Past and the Future Is Related to State Dissociation Among Individuals With High Levels of Psychopathological Symptoms. Frontiers in Psychology.

[17] Márquez Manel, Seguí Juan, García Luis, Canet Jaume, Ortiz Mercedes (2001). Is Panic Disorder with Psychosensorial Symptoms (Depersonalization-Derealization) a More Severe Clinical Subtype?. The Journal of Nervous and Mental Disease.

[18] Michal Matthias, Adler Julia, Wiltink Jörg, Reiner Iris, Tschan Regine, Wölfling Klaus, Weimert Sabine, Tuin Inka, Subic-Wrana Claudia, Beutel Manfred E., Zwerenz Rüdiger (2016). A case series of 223 patients with depersonalization-derealization syndrome. BMC Psychiatry.

[19] Sierra M., David A. S., Hunter E. C. M. (2004). The epidemiology of depersonalisation and derealisation. Social Psychiatry and Psychiatric Epidemiology.

[20] Simeon Daphne (2004). Depersonalisation Disorder. CNS Drugs.

[21] Lambert Michelle V., Sierra Mauricio, Phillips Mary L., David Anthony S. (2002). The Spectrum of Organic Depersonalization. The Journal of Neuropsychiatry and Clinical Neurosciences.

[22] Kolev Ognyan I., Georgieva-Zhostova Spaska O., Berthoz Alain (2014). Anxiety Changes Depersonalization and Derealization Symptoms in Vestibular Patients. Behavioural Neurology.

[23] Bertule Marika, Sebre Sandra B., Kolesovs Aleksandrs (2021). Childhood abuse experiences, depression and dissociation symptoms in relation to suicide attempts and suicidal ideation. Journal of Trauma &amp; Dissociation.

[24] (2023). Penn Study Finds Serotonin Reduction Causes Long COVID Symptoms.. Penn Medicine.

[25] Jones Derek K., Knösche Thomas R., Turner Robert (2013). White matter integrity, fiber count, and other fallacies: The do's and don'ts of diffusion MRI. NeuroImage.

[26] Sierra Mauricio, Nestler Steffen, Jay Emma-Louise, Ecker Christine, Feng Yue, David Anthony S. (2014). A structural MRI study of cortical thickness in depersonalisation disorder. Psychiatry Research: Neuroimaging.

[27] Daniels Judith K., Gaebler Michael, Lamke Jan-Peter, Walter Henrik (2015). Grey matter alterations in patients with depersonalization disorder: a voxel-based morphometry study. Journal of Psychiatry and Neuroscience.

[28] Scalabrini Andrea, Mucci Clara, Esposito Rosy, Damiani Stefano, Northoff Georg (2020). Dissociation as a disorder of integration – On the footsteps of Pierre Janet. Progress in Neuro-Psychopharmacology and Biological Psychiatry.

[29] Ning Yanzhe, Song Nan, Zhu Hong, Zheng Sisi, Jia Yuan, Yin Dongqing, Li Kuangshi, Jia Hongxiao (2022). White matter abnormalities in first-episode patients with depersonalization/derealization disorder: A tract-based spatial statistics study. Journal of Affective Disorders.

[30] Murphy Rachael J (2023). Depersonalization/Derealization Disorder and Neural Correlates of Trauma-related Pathology: A Critical Review.. Innovations in clinical neuroscience.

[31] Heydrich Lukas, Marillier Guillaume, Evans Nathan, Seeck Margitta, Blanke Olaf (2019). Depersonalization‐ and derealization‐like phenomena of epileptic origin. Annals of Clinical and Translational Neurology.

[32] Simeon Daphne, Guralnik Orna, Hazlett Erin A., Spiegel-Cohen Jacqueline, Hollander Eric, Buchsbaum Monte S. (2000). Feeling Unreal: A PET Study of Depersonalization Disorder. American Journal of Psychiatry.

[33] Büetiger Jessica R., Hubl Daniela, Kupferschmid Stephan, Schultze-Lutter Frauke, Schimmelmann Benno G., Federspiel Andrea, Hauf Martinus, Walther Sebastian, Kaess Michael, Michel Chantal, Kindler Jochen (2020). Trapped in a Glass Bell Jar: Neural Correlates of Depersonalization and Derealization in Subjects at Clinical High-Risk of Psychosis and Depersonalization–Derealization Disorder. Frontiers in Psychiatry.

[34] Krause-Utz Annegret, Frost Rachel, Winter Dorina, Elzinga Bernet M. (2017). Dissociation and Alterations in Brain Function and Structure: Implications for Borderline Personality Disorder. Current Psychiatry Reports.

[35] (2023). Depersonalization/Derealization Disorder - Psychiatric Disorders.

[36] (2023). Depersonalization/Derealization Disorder - Psychiatric Disorders.

[37] Simeon D., Abugel J. (2006). Feeling Unreal: Depersonalization Disorder and the Loss of the Self.

[38] Hunter E.C.M., Phillips M.L., Chalder T., Sierra M., David A.S. (2003). Depersonalisation disorder: a cognitive–behavioural conceptualisation. Behaviour Research and Therapy.

[39] Phillips Mary L., Sierra Mauricio (2003). Depersonalization Disorder: A Functional Neuroanatomical Perspective. Stress.

[40] Sierra Mauricio, Berrios German E (1998). Depersonalization: neurobiological perspectives. Biological Psychiatry.

